# Assessing vulnerability of densely populated areas to air pollution using Sentinel-5P imageries: a case study of the Nile Delta, Egypt

**DOI:** 10.1038/s41598-023-44186-4

**Published:** 2023-10-13

**Authors:** Mahmoud A. Hassaan, Salwa M. Abdallah, El-Sayed A. Shalaby, Amir A. Ibrahim

**Affiliations:** 1 Department of Environmental Studies, Institute of Graduate Studies and Research (IGSR), Alexandria University, Alexandria, Egypt; 2 Center of Excellence for Toxicological Testing, Central Agricultural Pesticides Lab (CAPL), Agricultural Research Center (ARC), Dokki, Giza, Egypt; 3 Soil and Water Department, Faculty of Agriculture, Alexandria University, Alexandria, Egypt

**Keywords:** Ecology, Environmental sciences, Environmental social sciences

## Abstract

Air pollution represents one of the major environmental stressors with serious implications on human health and ecosystem health. Recently remote sensing imageries; as an alternative cost and time-effective method compared with regular monitoring techniques, were used for provision of appropriate data concerning air quality over large areas. In this context, Sentinel-5P satellite provides high-resolution images of atmospheric pollutants including nitrogen dioxide, ozone, carbon monoxide (CO) and particulate matter (PM). The current work aims to delineate vulnerability of densely populated areas in Northern-Egypt to air pollution through retrieving CO and PM_2.5_ from Sentinel-5P images and validate the retrieved data through simultaneous In-Situ measurements. For this purpose, our approach comprised four-step methodology; data acquisition on study area, data manipulation, validation of retrieved air quality data and mapping the vulnerability to air pollution. Based on the data retrieved from the imagery, a composite vulnerability index for each CO and PM_2.5_ value was developed delineating the most vulnerable areas to air pollution in the Northern Nile Delta region. Such results revealed that Sentinel-5P imagery can serve as a valuable tool for monitoring air quality and assessing vulnerability of densely populated areas to air pollution. Accordingly, it can be concluded that the applied Sentinel-5P based model can be applied effectively for other air pollutants and can be extrapolated to other areas with similar and/or different environmental settings.

## Introduction

Massive quantities of emissions due to rapid economic development, urbanization, energy consumption, transportation and motorization, result in elaboration of a variety of pollutants into the atmosphere causing air pollution with significant negative implications on ecosystem health. These pollutants include, but not limited to particulate matter (PM_2.5_, PM_10_), sulfur dioxide (SO_2_), nitrogen dioxide (NO_2_), ozone (O_3_), carbon mono and dioxide (CO, CO_2_)^1^. In this respect, it was estimated that about 67 million tons of pollution were emitted into the atmosphere in the United States in 2021^2^. Worldwide, the harmful effects of air pollution on human health have been addressed including respiratory, cardiovascular and fertility diseases^3–6^ affecting all age groups particularly the vulnerable including children, women and elderly^7^. Furthermore, the World Health Organization (WHO) has been reported that air pollution represents the largest environmental health risk in the world where about 99% of the world’s population lives in places where the air pollutants exceed the WHO air guidelines levels^8^. Air pollution is also responsible for the greatest number of deaths, causing 6∙7 million deaths globally in 2019^9^.

Additionally, Air pollution has significant adverse impacts on both crop productivity and building materials. For instance, the aggregated damage cost caused by emissions was estimated as a range from 277 to 433 billion € in 2017^10^. Therefore, air pollution is considered as one of the most important and vital challenges, particularly in developing countries.

Addressing the challenge of CO emission into the atmosphere, through incomplete combustion of compounds such as gasoline, natural gas, oil, coal, and wood in addition to traffic emissions, industrial production, and biomass burning^11^, represents a prerequisite for evaluation of both air quality and potential risk to community health. Infants, elderly and people with anemia and/or a history of heart or respiratory diseases, are most likely to experience health effects upon exposure to elevated levels of CO^12^. CO can be considered also as a short-lived climate forcing agent since it contributes indirectly to climate change through participation in chemical reactions in the atmosphere that produce ozone; a climate change gas. Hence, prompting CO emission reductions is considered a possible strategy to mitigate effects of global warming.

On the other hand, PM represents a mixture of solid and/or liquid particles suspended in air resulting from smoke and dust and contains a higher number of harmful compounds. PM_10_ is considered as major component of air pollution and has threatened both environmental and human health whereas PM_2.5_ can cause cardiovascular, respiratory diseases and even early death^13^.

Deploying dense networks of instruments for air quality measurements are often too expensive and infeasible. Accordingly, there is a need for continuous spatio-temporal monitoring of air quality using In Situ measurements that are usually representative over a small spatial scale and provide only information at few selected points. In Situ measurements need a robust spatial and temporal modeling in order to detect the most affected areas in time (day, month, season)^14,15^.

Recently, remote sensing data can be used as a valuable alternative tool for monitoring air pollution and can support decision makers as they can be employed in retrieving and mapping of air quality parameters in a synoptic and multi-temporal coverage at regular intervals and dynamic scale^16,17^. The use of remote- sensed data in monitoring air pollution was promoted by the development of the space instruments designed to observe air pollutants, starting with the launch of the Total Ozone Monitoring Instrument (TOMS) in 1978, the Global Ozone Monitoring Experiment (GOME) in 1995, and Ozone Monitoring Instrument (OMI) in 2004^18^.

Sentinel-5P TROPOMI is the latest satellite-based sensor that provided new opportunities for utilizing remote- sensed data in monitoring air quality^19,20^. Sentinel-5P TROPOMI covers wavelength ranges between ultraviolet and short-wave infrared, enabled observations of different species of air pollutants such as CO, NO_2_, O_3_, SO_2_, CH4, HCHO, and Absorbing or Ultra Violet Aerosol Index (AAI or UVAI), from which surface particulate matter (PM_2.5_ and PM_10_) may be inferred^17^. Sentinel-5P TROPOMI has a relatively high spatial resolution (3.5 * 7 km^2^ for all trace gases, except for CO and CH_4_ that is 7 * 7 km^2^)^16,21^.

The research in hand is intended to delineate vulnerability of densely populated areas to air pollution through retrieving CO, and PM_2.5_ from Sentinel-5P images and validating the retrieved data through simultaneous In Situ measurements.

## Materials and methods

### Study area

**Figure 1:**
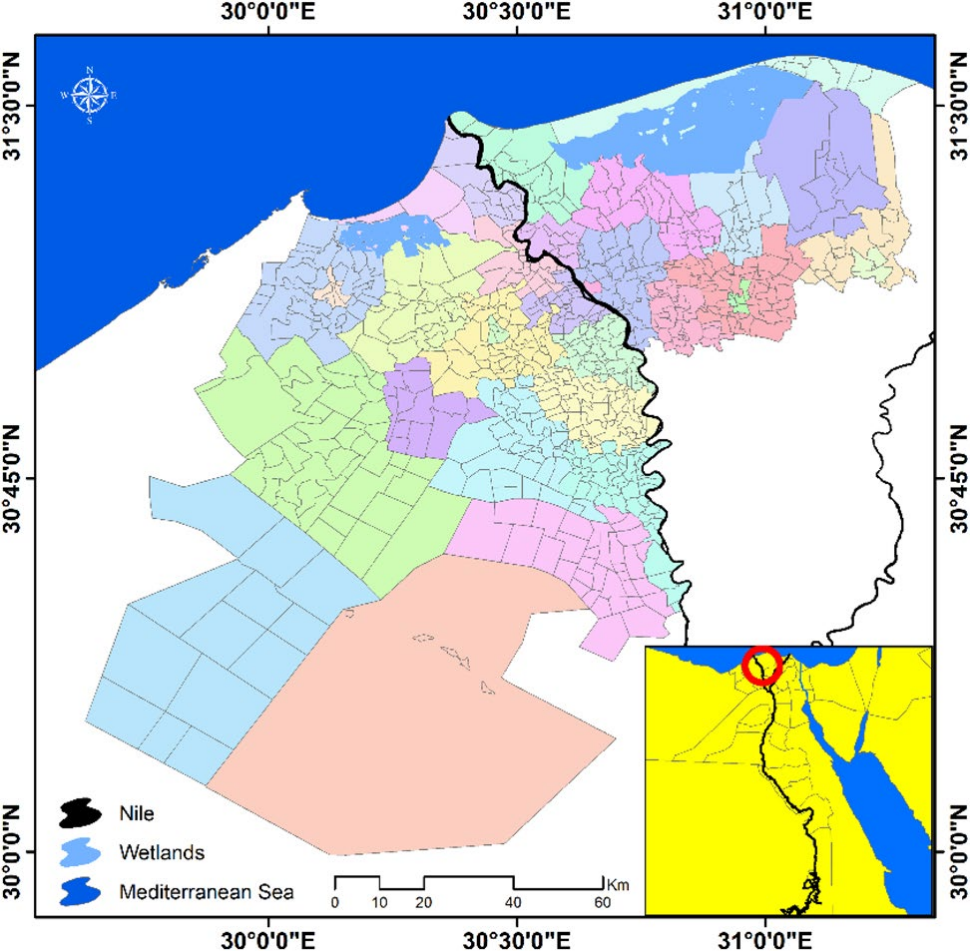
Study area and administrative subdivisions. *Source*: developed by the authors using ArcGIS (Ver. 10.8).

The study area includes two coastal governorates of the Nile Delta region; Kafr El Sheikh and El Behira Governorates covering a total area about 15,000 km^2^ and divided administratively into 32 districts, which are further subdivided into 765 Localities (Fig. 1). The total population of both governorates was estimated recently to be 10.2 million in 2021, which represents about 10% of the total Egyptian population^22^. Thus the study area represents a densely populated area with a variety of economic activities including agriculture, manufacturing and aquaculture.

### Methodology

**Figure 2:**
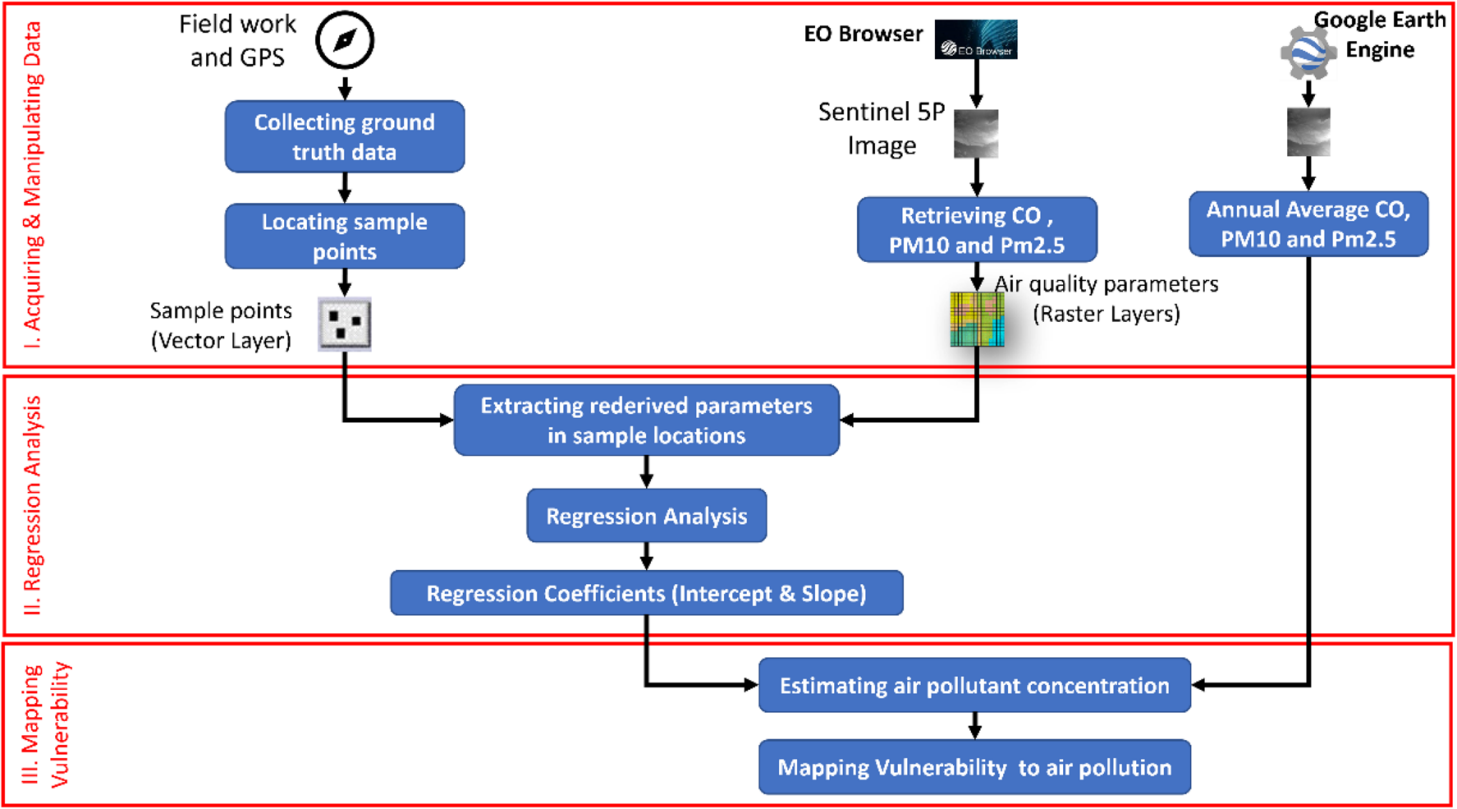
Mythology of mapping vulnerability to air pollution through remote-sensed data.

In order to assess vulnerability of densely populated areas in the Nile Delta region to air pollution through Sentinel-5P remotely sensed data, a methodology of four main steps was applied including: acquiring and manipulating data, validating retrieved air quality data, and mapping vulnerability to air pollution (Fig. 2).

#### Acquiring and manipulating data

**Figure 3:**
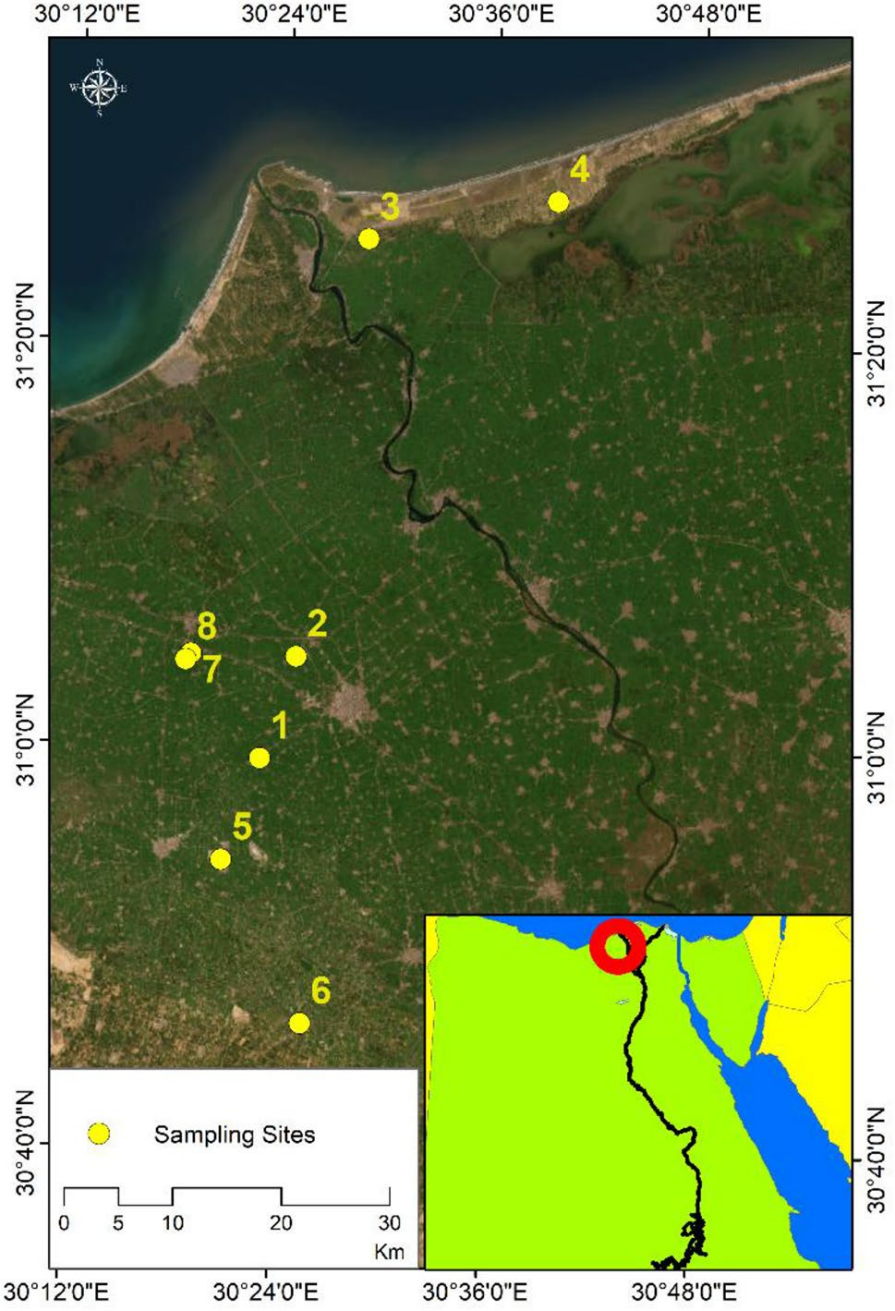
Samples sites of CO concentrations for In-Situ measurements on World Imagery ArcGIS Online Base map. *Source*: developed by the authors using ArcGIS (Ver. 10.8)^25^.

**Figure 4:**
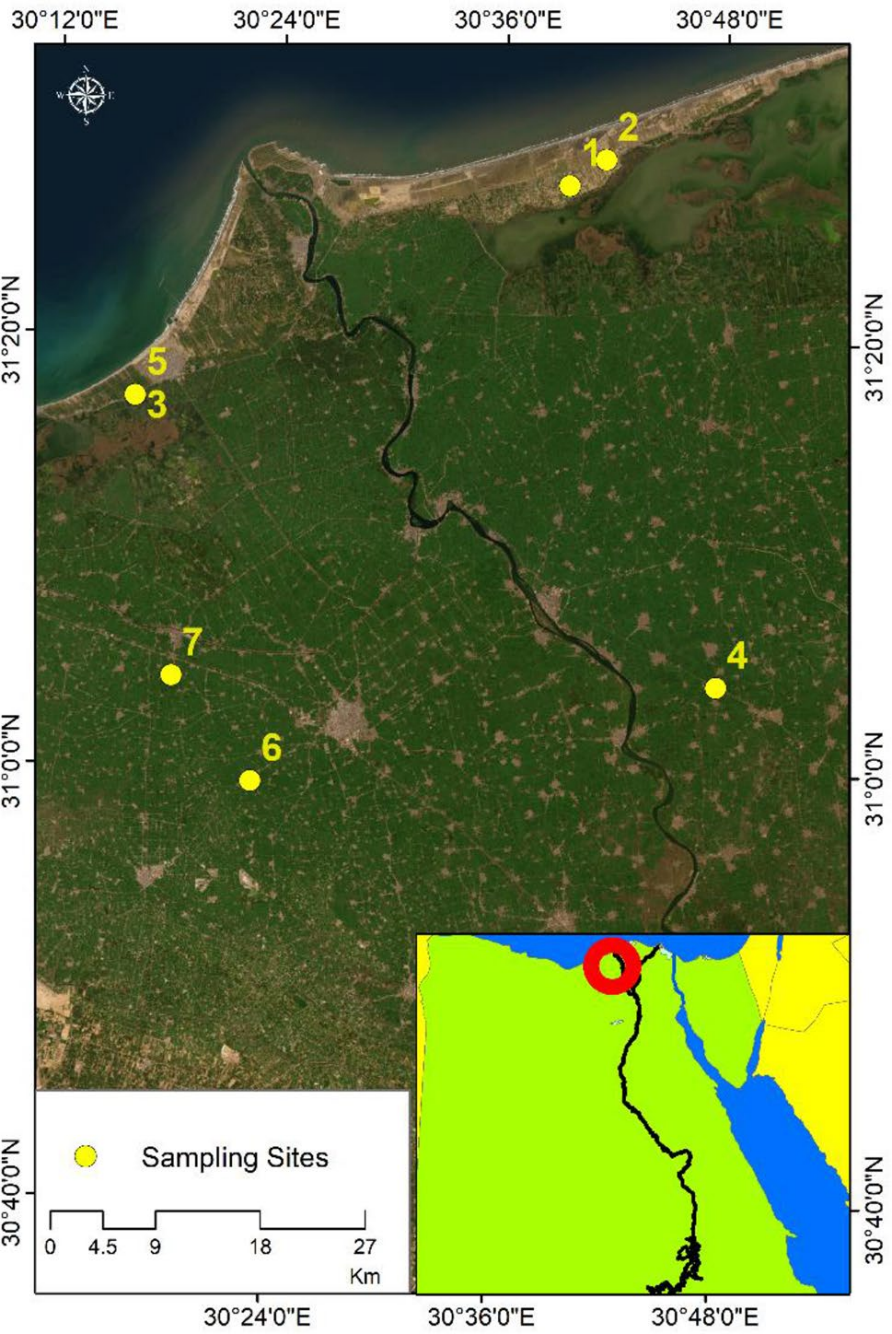
Samples sites of PM concentrations for In-Situ measurements on world imagery ArcGIS Online base map. *Source*: developed by the authors using ArcGIS (Ver. 10.8)^25^.

This step involves acquiring ground-truth data that were gathered through four field trips undertaken during the period from October 2020 to June 2021. A number of ground truth georeferenced measurments were carried out at different locations within the study area (Tables 1, 2 and Figs. 3, 4). Measurement of CO concentrations was conducted using standard test method for Carbon Monoxide in the Atmosphere through CO automatic continuous monitor (Continuous Measurement by Nondispersive Infrared Spectrometry). Also, (EVM7) was employed to measure the particulate matter; PM_10_ and PM_2.5_ using both the gravimetric method and the laser scattering theory.

**Table 1:** CO concentration in air samples collected from different sites.

Site	Longitude	Latitude	Directorate	Date	CO (mg/m^3^)
1	30.382	30.989	Damanhur	07/10/2020	78
2	30.415	31.073	Damanhur	07/10/2020	5
3	30.476	31.420	Motobas	13/10/2020	38
4	30.658	31.454	Motobas	13/10/2020	47
5	30.348	30.905	Housh Eissa	09/06/2021	8
6	30.427	30.770	Housh Eissa	09/06/2021	29
7	30.314	31.074	Abu Hommos	09/06/2021	31
8	30.309	31.069	Abu Hommos	09/06/2021	32

**Table 2:** PM concentration in air samples collected from different sites.

Site	X	Y	Directorate	Date	PM_10_ (mg/m^3^)	PM_2.5_ (mg/m^3^)
1	30.309	31.069	Abu Hommos	07/10/2020	61.250	21.510
2	30.658	31.454	Motobas	13/10/2020	9.910	0.920
3	30.691	31.474	Motobas	13/10/2020	20.520	13.620
4	30.271	31.285	Edku	13/10/2020	36.410	16.810
5	30.799	31.068	Qleen	11/04/2021	44.320	21.480
6	30.271	31.285	Edku	11/04/2021	29.560	19.020

Additionally, this step includes acquiring satellite-based observations for CO concentration and UVAI. In this respect, two datasets for air quality parameters in the study area via Sentinel-5P imageries were collected. The first dataset involved simultaneous data on CO column number density measured in the unit of mol/m^2^ and UVAI, which is unitless, were collected from Sentinel Hub EO Browser^23^. EO Browser allows downloading satellite data from numerous satellites and data collections instantly. This dataset was used to calibrate the relationships between satellite-based observations and ground-truth data through regression analysis.

Meanwhile, the second dataset comprised average CO column number density and UVAI over the study area during the period (1 October 2020–30 June 2021). For this purpose, the Google Earth Engine; an Internet-based platform that provides satellite imagery and cloud-based computing algorithms^24^, was employed to download such data. These datasets was further employed to retrieve average CO and PM concentrations in the study area during the study period.

#### Regression analysis

To investigate the relationship between CO and UVAI concentrations retrieved from Sentinel- 5P images and In-Situ measurement, a regression analysis was applied. For that purpose, various georeferenced sampling sites were allocated and employed for extracting the retrieved CO, PM_10_ and PM_2.5_ at the corresponding locations of the measurements. This was carried out through Spatial Analyst in ArcGIS (Extract Values to Points Tool).

Thereafter, relationships between CO concentration retrieved from Sentinel- 5P images as a dependent variable and ground-truth data of CO concentration was estimated through linear regression analysis. Similarly, the relationship between Aerosol Index and the ground truth data of PM_10_ and PM_2.5_ was investigated.

#### Mapping vulnerability to air pollution

Finally, the slope and intercept values resulted from regression analysis were applied to air quality datasets downloaded from Google Earth Engine for estimating the average concentration of air quality parameters in various parts of the study. This is followed by mapping various air quality parameters and assessing vulnerability of air pollution. For this purpose, the average concentrations of CO and PM_2.5_ were estimated for each locality in the study area through Zonal Statistics Tool in ArcGIS.

Thereafter, two composite vulnerability indexes were developed for CO and PM_2.5_ considering two primary indicators namely; pollutant concentration prevailed in each locality and population size exposed to the pollutant. Therefore, a direct-relationship can be established since localities that have high pollutant concentration and large population size have been exposed to such pollutant compared to those having low concentration of pollutant and small population size.

The primary indicators were normalized through the following formula:

$${N}_{x}=\frac{X-Xmin }{Xmax- Xmin}$$

where ${N}_{x}$, Normalized value *of primary indicator*; *X*, Absolute value of primary indicator; ${X}_{min}$, Minimum absolute value of primary indicator; ${X}_{max}$, Maximum absolute value of primary indicator.

Thereafter, the composite vulnerability index was calculated through aggregating normalized primary indicators for each pollutant assuming equal weights of the three indicators, according to the following formula:

$$V = {\sum }_{ i=1}^{n} \left( {N}_{i} * {W}_{i}\right)$$

where V, Vulnerability index; *N*_*i*_, Normalized value of indicator *i*; W_*i*_, Weight of indicator N_*i*_

It should be noted that the resulting composite index is a relative index ranging between 0 for least vulnerable localities and 1 for most vulnerable localities.

## Results and discussion

**Figure 5:**
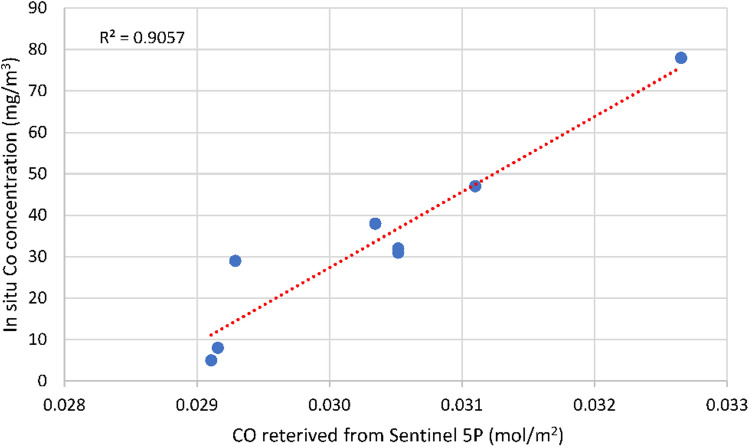
Linear relationship between CO concentration retrieved from Sentinel-5P and In-Situ CO concentration.

**Figure 6:**
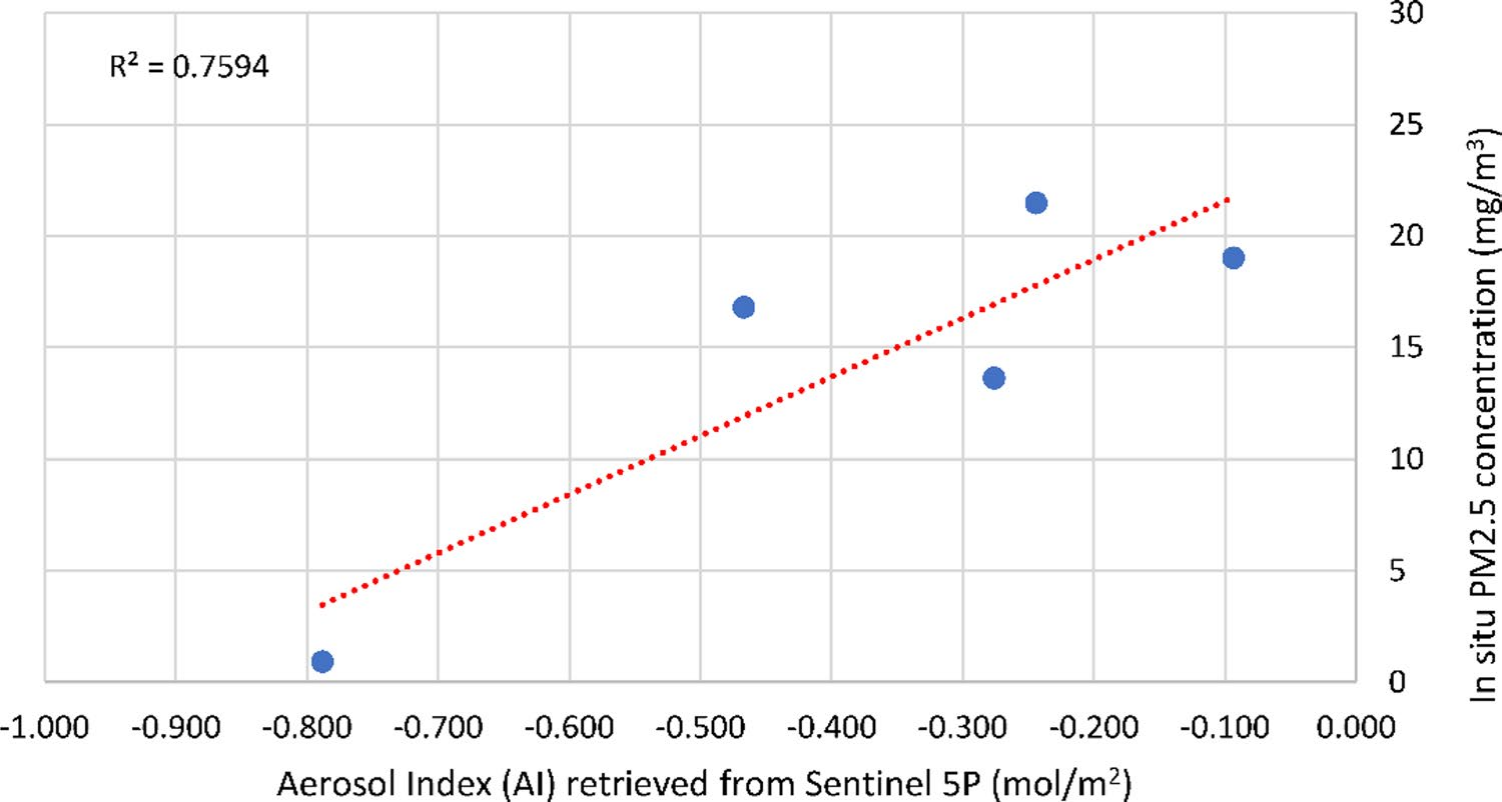
Linear relationship between Aerosol Index retrieved from Sentinel-5P and In-Situ PM_2.5_ concentration.

As a result of regression analysis, strong relationships between ground-truth data of CO concentrations and CO retrieved from Sentinel-5P images with R^2^ = 0.91 (*P* value < 0.01) (Fig. 5) were recorded. Similarly, ground-truth data of PM_2.5_ concentration was found to have strong relationship with Aerosol index recording R^2^ of 0.79 (*P* value < 0.05) (Fig. 6). Meanwhile, the relationship between PM_10_ and Aerosol Index was found to be insignificant (Table 3).

**Table 3:** Regression analysis of ground-truth and retrieved data.

Independent Variable	CO (mg/m^3^)	PM_10_ (mg/m^3^)	PM_2.5_ (mg/m^3^)
Dependent Variables	CO (mol/m^2^)	Aerosol Index	Aerosol Index
Multiple R	0.952	0.653	0.890
R Square	0.906	0.426	0.792
Adjusted R Square	0.890	0.282	0.740
Standard Error	7.599	15.324	3.963
Observations	8	6	6
Intercept	− 519.557	48.854	24.463
Slope	18,231.040	45.603	26.725
*P* value	0.0003	0.160	0.0175

This means that remote- sensed CO concentration and Aerosol Index are effective in predicting CO and PM_2.5_ concentrations in mg/m^3^. Based on the intercept and slope derived from regression analysis, the concentration of CO and PM_2.5_ can be estimated through the following formula:

$${\text{y}} = 18231{\text{x}} - 519.56$$

where y, CO Concentration (mg/m^3^); x, CO Concentration retrieved from Sentinel-5P (mol/m^2^).

Also, PM_2.5_ concentration can be estimated according to the following formula:

$${\text{y}} = 26.725{\text{x}} + 24.463$$

where: y, PM_2.5_ Concentration (mg/m^3^); x, Aerosol Index retrieved from Sentinel-5P.

**Figure 7:**
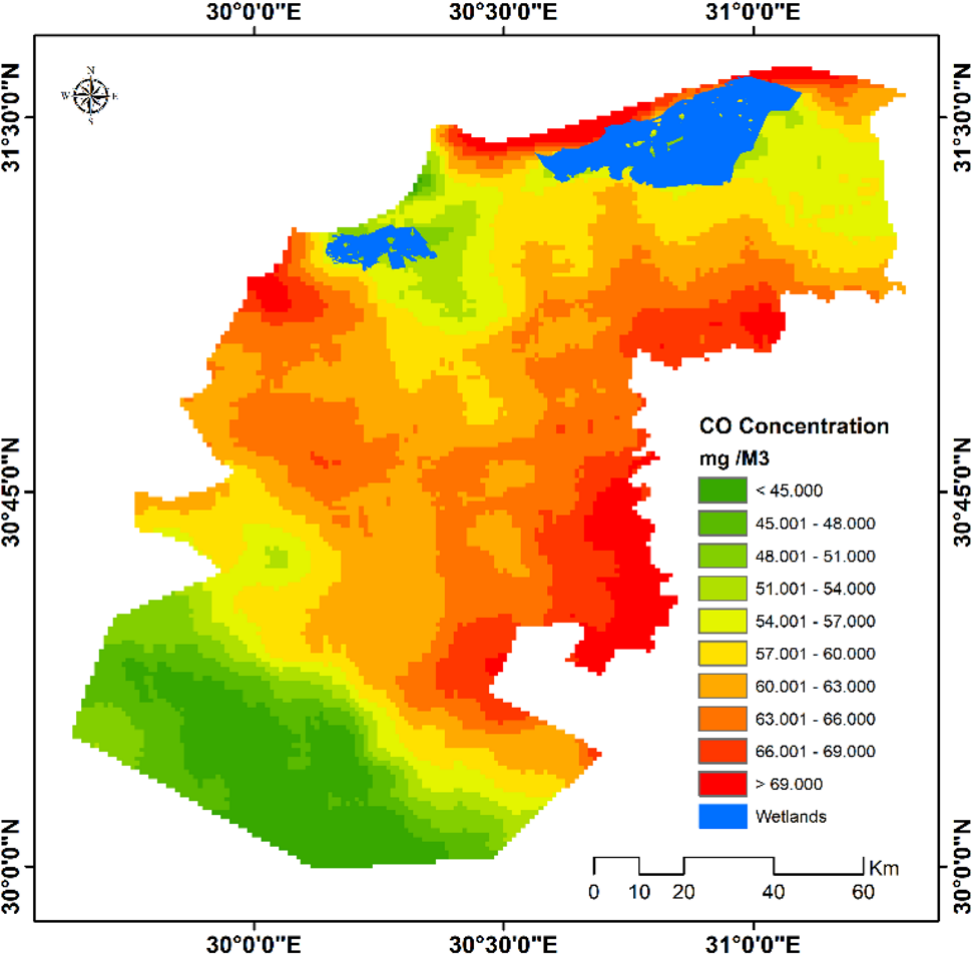
Average CO concentration in the study area (October 2020–June 2021). *Source*: developed by the authors using ArcGIS (Ver. 10.8).

The estimated average CO concentration during the period (October 2020–June 2021) revealed that different parts of the study area has experienced varied level of CO concentration ranging between 40–78 mg/m^3^. The highest CO concentrations were noted as some spots in the northern, western, and southern parts the study area. Meanwhile, the south-western parts of the study area have experienced lowest level of CO (Fig. 7).

It is worthy to mention that retrieved data concerning CO concentrations in the study area seem to be overestimated. However, such an overestimation can be overlooked as that the suggested methodology in this study for delineating vulnerability to air pollution is dependent on a composite index, which provides a relative measure, rather than absolute one, ranging between 0 and 1 reflecting for least and most level of vulnerability; respectively.

**Figure 8:**
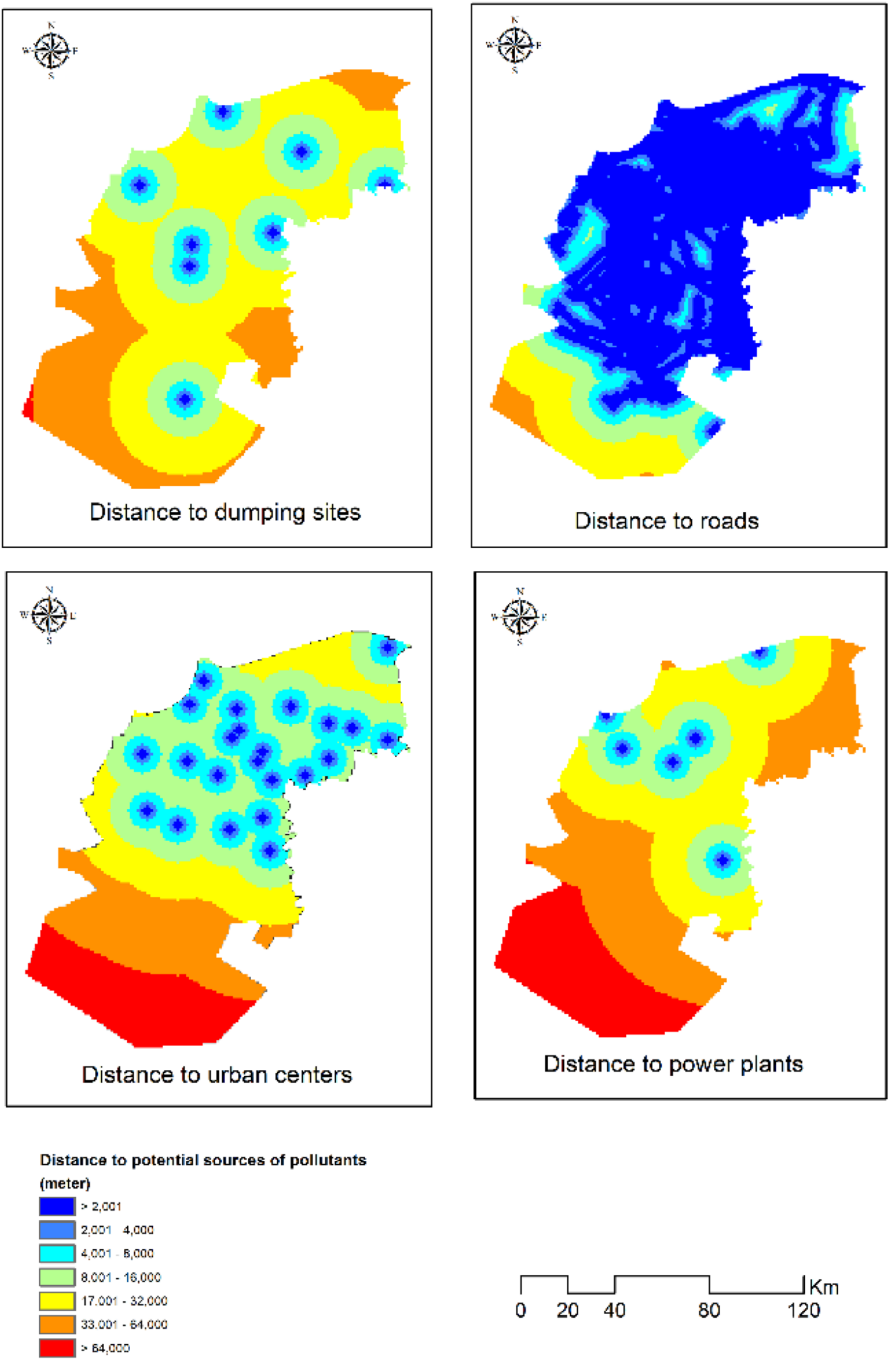
Distance to potentials sources of CO emission. *Source*: developed by the authors using ArcGIS (Ver. 10.8).

To explain such a spatial pattern of CO concentration, correlation coefficients between CO concentration in the study area and potential sources of pollution such as closeness to dumping sites, power plants, highways and urban centers were estimated using Multivariate Analysis Tools (Fig. 8). Our results exhibited that CO concentration has strong negative correlation with distance to highways (− 0.722), to power plants (− 0.684) and to urban centers (-0.689); respectively. Such findings clarified that the concentration of CO decreases notably with increasing distance to power plants, highways and urban centers as potential sources of CO emission. Meanwhile, CO concentration was found to have a moderate negative correlation with distance to dumping sites (− 0.412) (Table 4).

**Table 4:** Correlation Coefficient between CO Concentration and key potential sources.

Variables	CO Concentration
Distance to highways	− 0.722
Distance to dumping sites	− 0.412
Distance to power plants	− 0.684
Distance to urban centers	− 0.689

**Figure 9:**
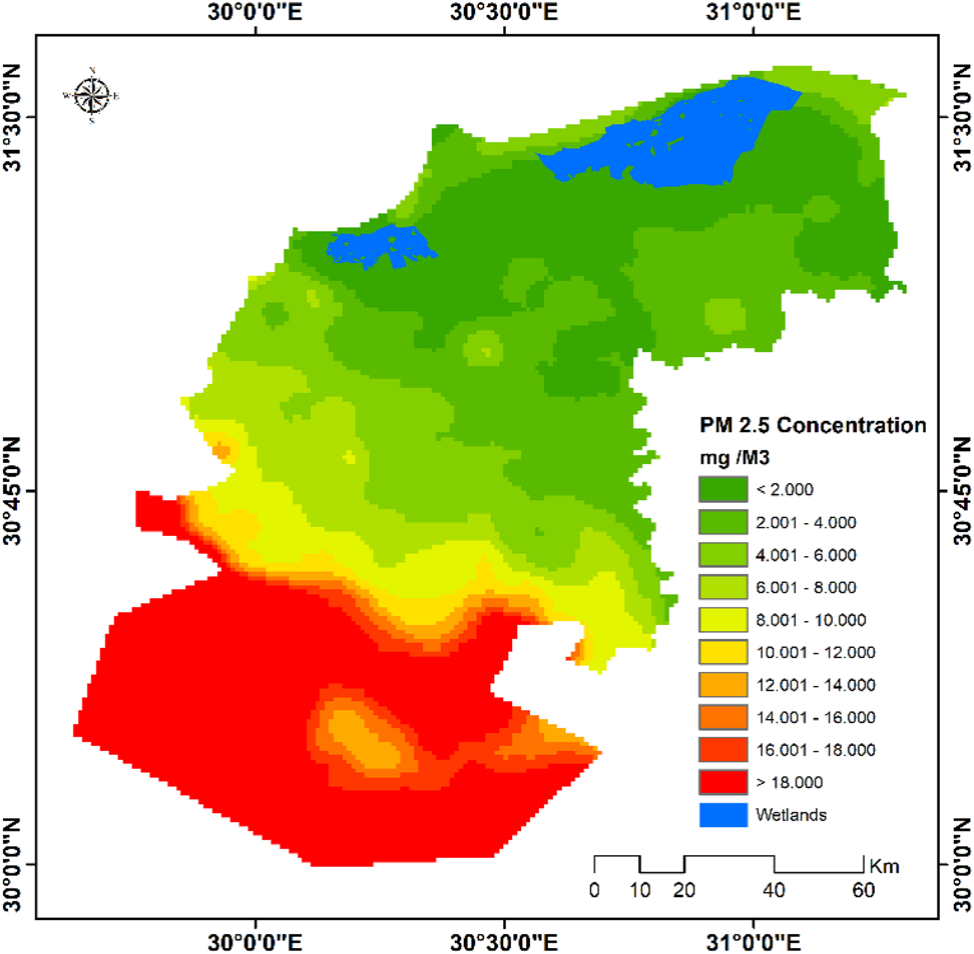
Average PM_2.5_ concentrations in the study area (October 2020–June 2021). *Source*: developed by the authors using ArcGIS (Ver. 10.8).

Furthermore, it was found that concentration range of PM_2.5_ in the study area was < 2 to 32 mg/m^3^ and PM_2.5_ concentrations decreased gradually from north-east to south-west, where the south-western parts recorded the highest concentrations (Fig. 9).

The results of the current study are consistent with previous studies in Egypt, which recorded highest levels of PM_2.5_ concentration in the western margin of the Nile Delta exceeding 1350 µg/m^3^ on average^25^ and association of highest levels of CO concentration with industrial areas, where CO concentration exceeded 3000 mg/m^3^^26^.

**Figure 10:**
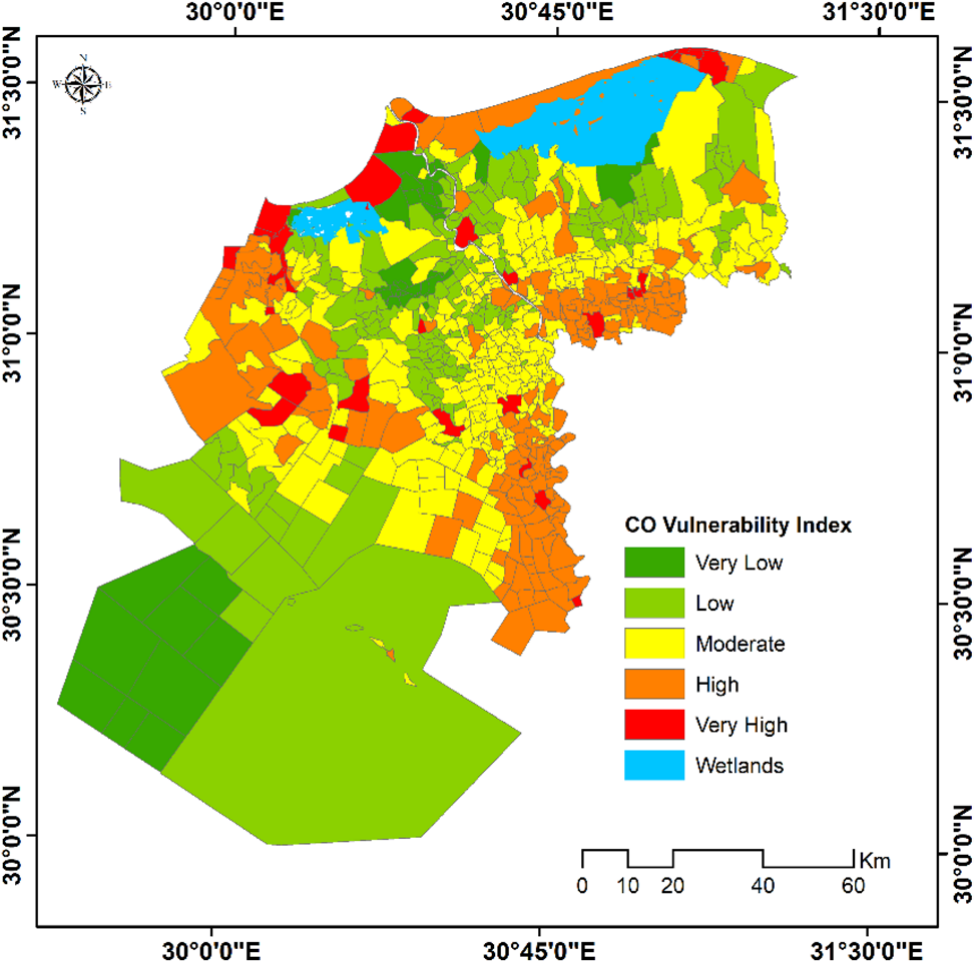
Vulnerability to CO (October 2020–June 2021). *Source*: developed by the authors using ArcGIS (Ver. 10.8).

Application of the composite index approach of vulnerability to air pollutants; mentioned earlier in the proposed methodology, revealed that different localities are susceptible to varied levels of CO and PM_2.5_. About 47.7% of total population in the study area was categorized as very highly to highly vulnerable to CO (average CO concentrations exceeded 65 mg/m^3^). Meanwhile, 31.1% of the population were moderately vulnerable (average CO concentrations were 63 mg/m^3^) and only 20.2% of the total population are living in low to very low vulnerability zones with an average CO concentration of 57 mg/m^3^ (Fig. 10).

**Figure 11:**
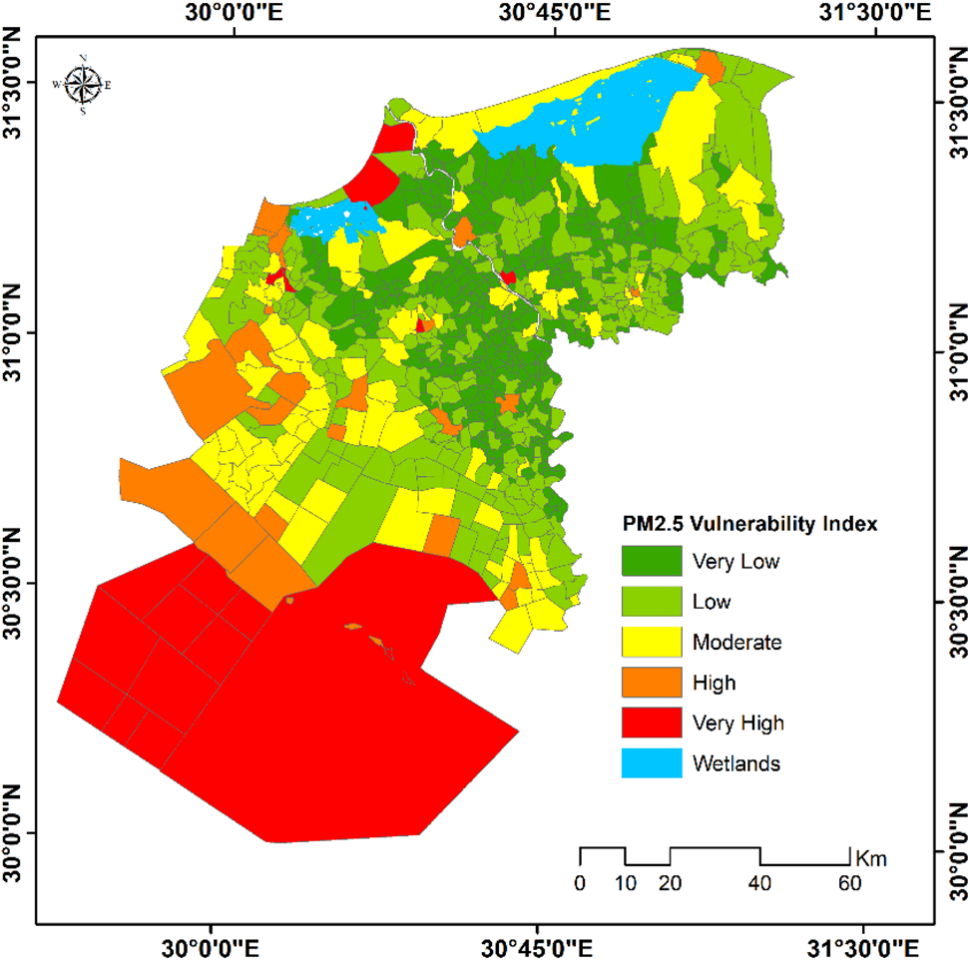
Vulnerability to PM_2.5_ (October 2020–June 2021). *Source*: developed by the authors using ArcGIS (Ver. 10.8).

Comparatively, the population of the study area was found to be less vulnerable to PM_2.5_, where about 19.6% of total population are living within high to very high vulnerability zones (average PM_2.5_ concentration recorded 13 mg/m^3^). Meanwhile, 22.6% of the population are moderately vulnerable to PM_2.5_ and 57.8% of the total population are living in low to very low vulnerability zone with an average PM_2.5_ concentration not exceeding 3 mg/m^3^ (Fig. 11).

## Conclusion

Remote-sensing data can serve as a cost effectiveness tool for monitoring air quality. In this regard, strong correlations were established in the current work between Sentinel-5P data, CO and PM_2.5_ ground-truth data as two of the major air pollutants. Sentinel-5P data were further employed for retrieving and mapping of CO and PM_2.5_ concentrations in two governorates of the Nile Delta region. Distribution pattern of CO concentrations in the study area was found to be associated with distance to main sources of CO emission.

Furthermore, using the retrieved parameters accompanied with the population size facilitated the determination of a composite vulnerability index for the pollutants of concern. The developed vulnerability index highlighted different localities in the study area with varied levels of vulnerability and identified the main hotspots.

It’s worth noting that using Sentinel-5P imageries for monitoring air quality requires availability of simultaneous In-Situ measurements for validation purposes. According to our case study results, it can be concluded that the implemented study model can be tested and applied for other air pollutants and can be extrapolated to other areas with similar or different environmental settings.

## Data Availability

The datasets used and/or analyzed during the current study available from the corresponding author on reasonable request.
